# Supplementary material for: Biogenic carbonate mercury and marine temperature records reveal global influence of Late Cretaceous Deccan Traps
Source: Nat Commun. 2019 Dec 16;10:5356. doi: 10.1038/s41467-019-13366-0 (PMC6915775; doi:10.1038/s41467-019-13366-0)
Supplement: Supplementary file 2 — Supplementary Information [file 41467_2019_13366_MOESM2_ESM.pdf]

Supplementary Information:
Biogenic Carbonate Mercury and Marine Temperature Records Reveal Global Influence of Late Cretaceous Deccan Traps

Kyle W. Meyer *et al.*

Supplementary Figures

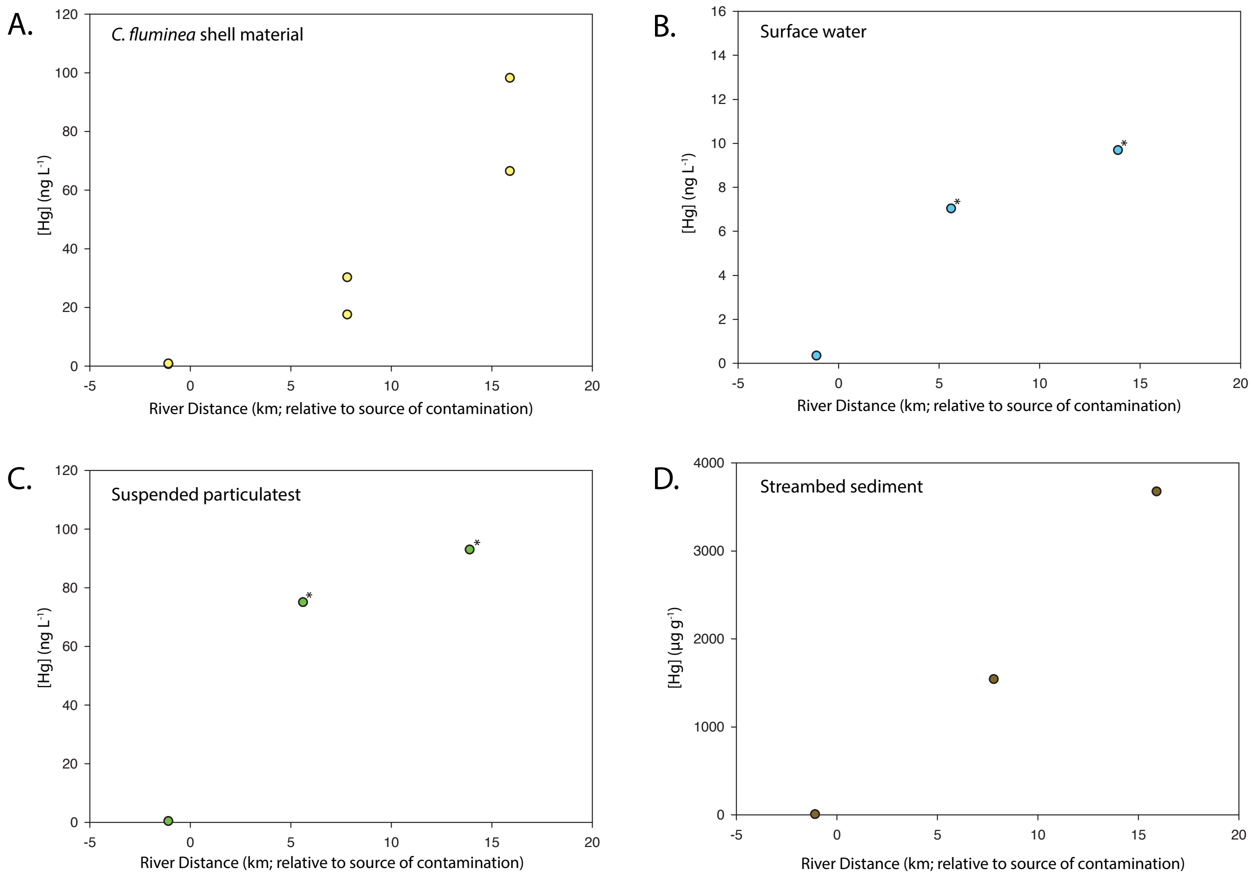

Supplementary Figure 1. [Hg] values of samples from the South River, Virginia, USA. Sample material includes *C. fluminea* shell material (this study)(A), and material collected by Washburn et al. (2017, 2018) including: Filtered surface water (B), suspended particulates (C), and streambed sediment (D). Data depicted with an '*' correspond to sampling from 2014 (Washburn et al., 2017) all other data was collected in 2016 (Washburn et al., 2018).

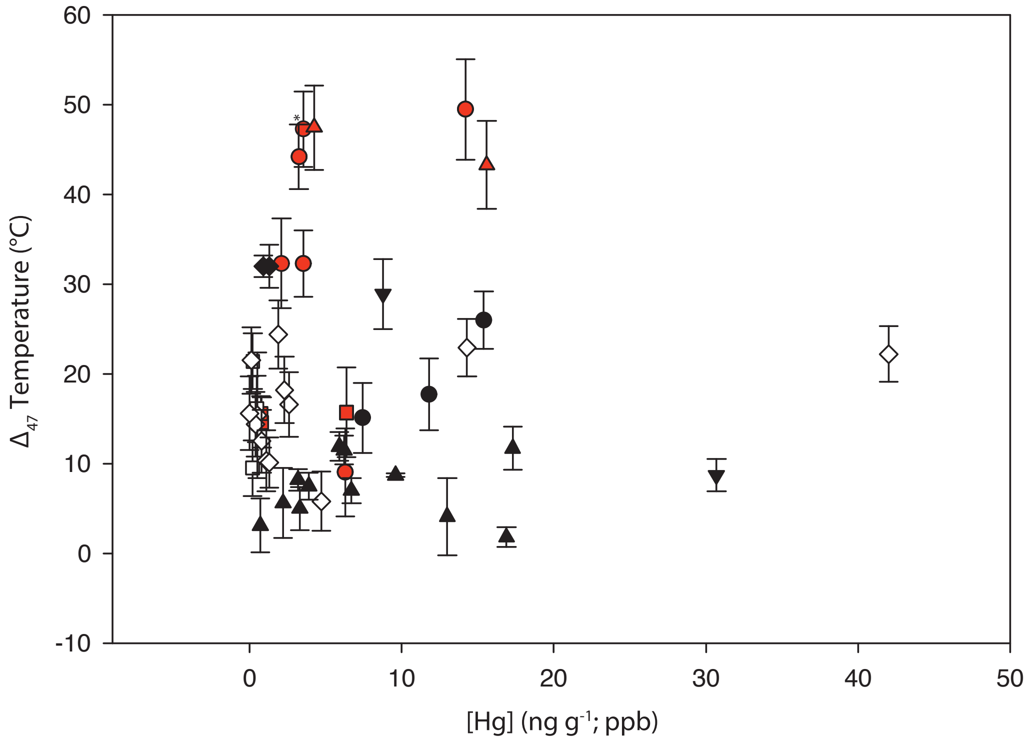

Supplementary Figure 2. Measured [Hg] versus calculated Δ_{47}-derived coastal marine temperatures by global locality. Samples colored red were classified as 'altered' and were determined to be likely susceptible by diagenetic alteration based on either Δ_{47}, $^{87}Sr/^{86}Sr$, or major/minor/trace element (De Winter, *pers. comm.*) compositions. Samples, by locality, are: Merced County, California (black circles & red circle with '*'); Mt. Katmai, Alaska (red circles); North Slope, Alaska (upside-down black triangles); San Juan Islands, Washington (red triangles); Neuquén Basin, Argentina (red squares); Scania, Sweden (white squares); Fezzan, Libya (black diamonds); Moscow Landing, Alabama (white diamonds); and Seymour Island, Antarctica (black triangles). Generally, outside of the Deccan Traps eruptive interval, no correlation is observed between temperature and [Hg], which would imply no significant temperature-dependent Hg incorporation/partitioning.

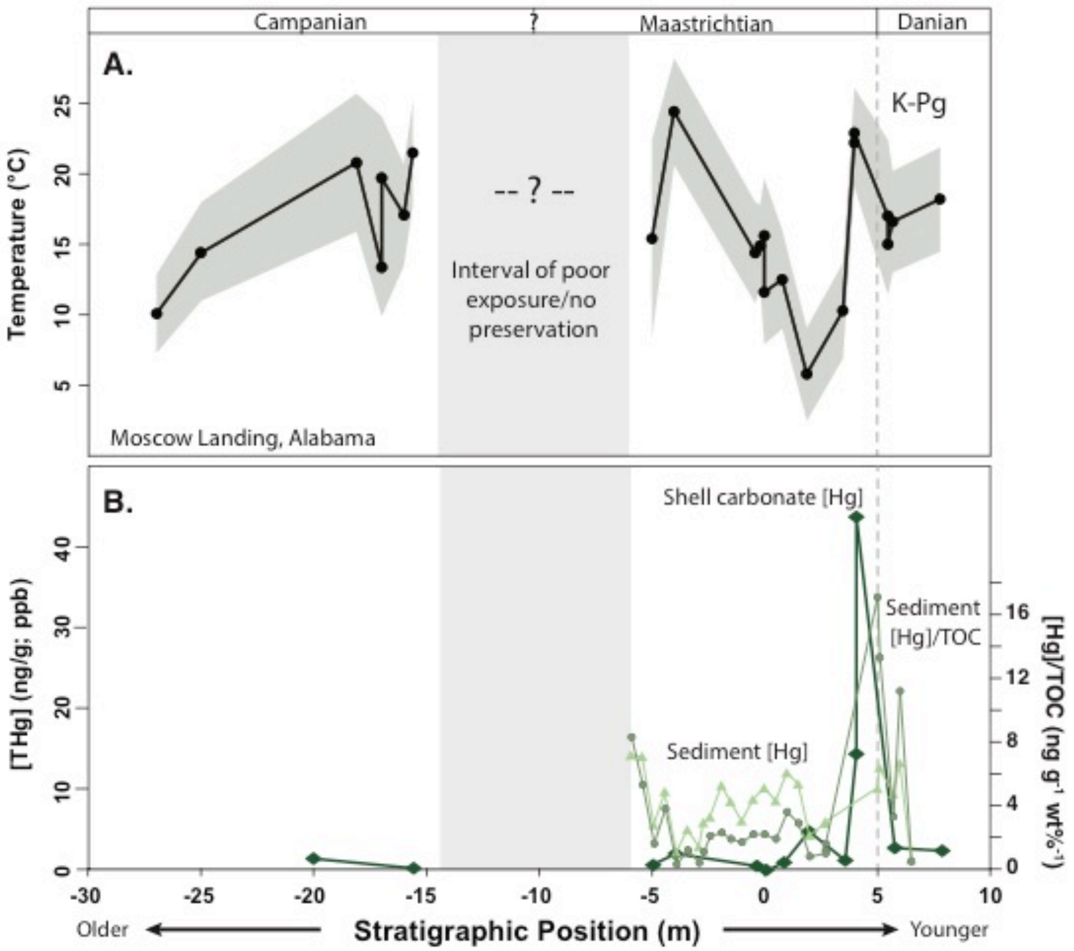

Supplementary Figure 3. Record of Late Maastrichtian fossil bivalve shell Δ_{47} (A; circles) and [Hg] (B; diamonds) compared to sediment [Hg] (B; triangles) and [Hg]/TOC (B; circles) at Moscow Landing, Alabama. Direct comparisons between shell carbonate and sediment [Hg] would imply a decoupling between these two archives, however, upon normalizing sediment [Hg] with respect to wt. % TOC in accordance with other published [Hg] studies[1-6] a pattern of similarly elevated Hg loading near the K-Pg boundary is revealed in both archives. Uncertainties on any single sample [Hg] value are, on average, ±11% of the measured [Hg] value (please refer to Methods).

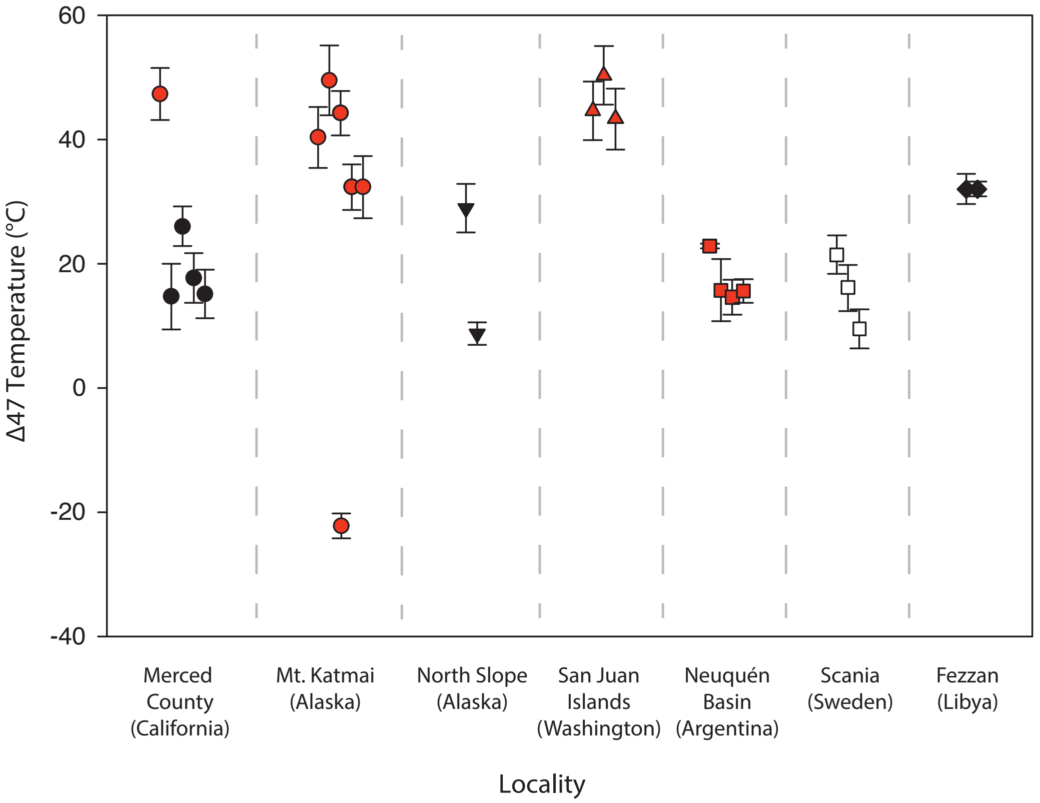

Supplementary Figure 4. Calculated Δ_{47}-derived coastal marine temperatures by global locality. Samples colored red were classified as 'altered' and were determined to be likely susceptible by diagenetic alteration based on either Δ_{47}, $^{87}Sr/^{86}Sr$, or major/minor/trace element (De Winter, *pers. comm.*) compositions. No temperature values exceeded 100 °C, which would suggest a very low likelihood of Hg mobilization.

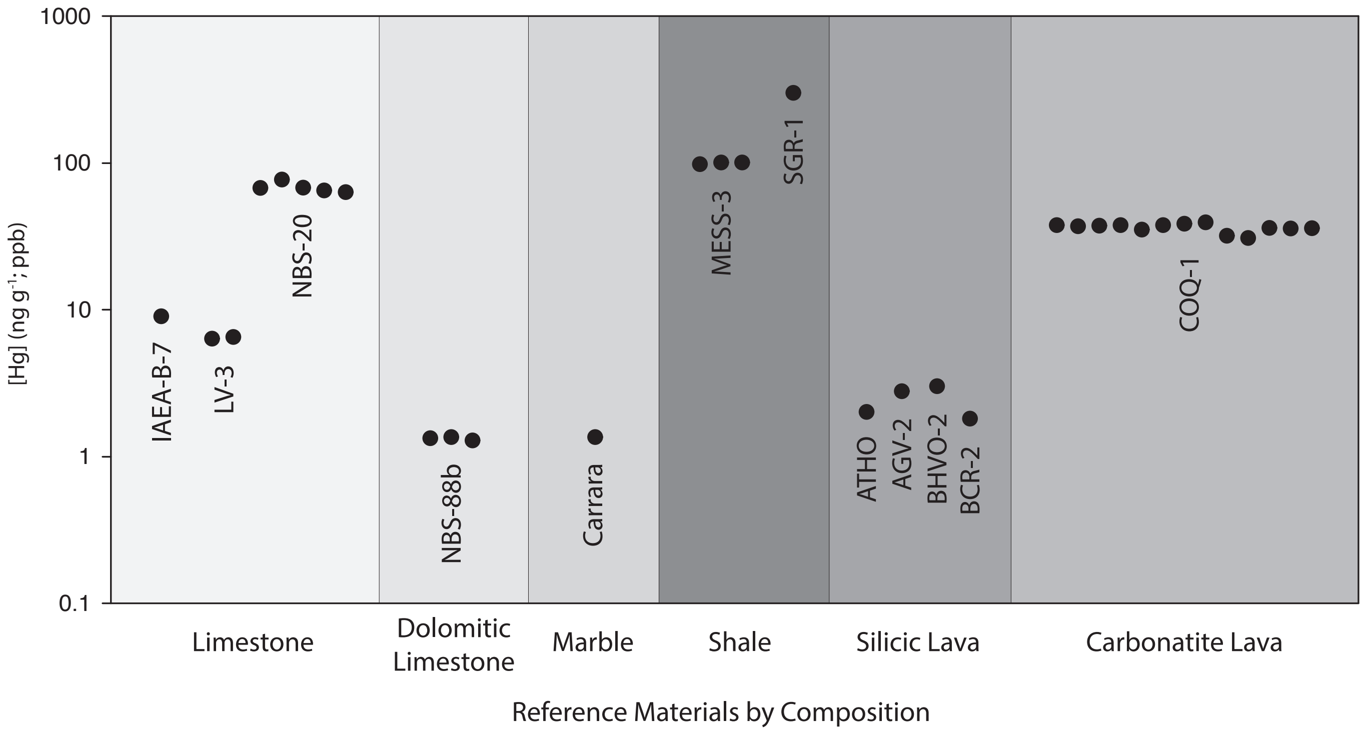

Supplementary Figure 5. [Hg] values of reference materials by geologic composition. In this study, we analyzed a suite of reference materials to propose a carbonate [Hg] standard. Of all the materials evaluated, USGS reference material COQ-1, a carbonatite lava sample, appeared to be the best-available candidate standard. Please refer to Table S5 and Discussion Section S10 for additional information regarding the reference materials and analyses. Uncertainties for these analyses were approximately ±11% of the measured [Hg] and are typically too small to depict.

Supplementary Table

Supplementary Table 1. *Modern and ancient geographic coordinates of sample locations*

Locality Identifier	Modern Latitude (°N)	Modern Longitude (°W)	Paleolatitude[†]
AF-KAG	58.92691	-153.9989	~62 °N[‡]
IL-KAG	59.01667	-153.96533	~62 °N[‡]
MK-KAG	58.88942	-154.05224	~62 °N[‡]
EL-SCH	69.40365	-151.90194	~83–85 °N[*]
OP-PRI	70.08333	-151.41667	~83–85 °N[*]
WI-NAN	48.71139	-123.00917	~30 °N[**]
SI-NAN	48.75023	-122.91	~30 °N[**]
MC-MOR	36.95583	-120.8775	~21 ± 5 °N[***]
SC-BAL	56.16611	14.35806	46 °N
DT-DAK	25.535	30.55444	8 °N
KO-DAK	25.48639	30.55278	8 °N
LI-FEZ	26.54778	12.73694	10.5 °N
IN-KAL	11.23778	79.29056	~35 °S
BJ-VES	-38.10292	-68.38903	~43 °S[****]

[†]Paleolatitude determined after Torsvik et al. (2012) via www.paleolatitude.org
[‡]Paleolatitude estimated in Pasch and May (1997).
[*]Paleolatitdue estimated by Salazar-Jaramillo et al. (2015).
[**]Paleolatitude estimated by Peecook and Sidor (2015).
[***]Paleolatitude estimated by Kodama and Ward (2001).
[****]Paleolatitude estimated by de Winter et al. (2017).

Supplementary Notes

Supplementary Note 1. *Sample Locality Information*

Locality Identifier: BJ-VES ("Baja de Jaguël", *vesicularis*) **Formation:** Jaguël

Samples collected here:

- BJ-VES-M4u: *Pycnodonte vesicularis* (Lamarck) collected at a position described as "ex situ, upper 8m"
- BJ-VES-M5u: *Pycnodonte vesicularis* (Lamarck) collected at a position described as "ex situ, upper 8m"
- BJ-VES-M8u: *Pycnodonte vesicularis* (Lamarck) collected at a position described as "upper 2m"
- BJ-VES-M10: *Pycnodonte vesicularis* (Lamarck) collected at a position described as "~50 cm below KPB"
- BJ-VES-M11u: *Pycnodonte vesicularis* (Lamarck) collected at a position described as "~50 cm below KPB"

Location: Baja de Jaguel section described in de Winter et al. (2017) and Woelders et al. (2017)
Collection site description: Refer to de Winter et al. (2017) and Woelders et al. (2017)
Collected by: de Winter et al. (2017)
Sample obtained from: Johan Vellekoop

Locality Identifier: IN-KAL ("India, Kallankurchchi") **Formation:** Kallankurchi

Samples collected here:

- IN-KAL-UNGa: *Agerostrea ungulata* (von Schlotheim)
- IN-KAL-UNGb: *Agerostrea ungulata* (von Schlotheim)

Location: Cauvery Basin, India
Collection site description: No stratigraphic details provided. Stratigraphic sections detailed in Nagendra et al. (2011) and Zakharov et al. (2011).
Collected by: R. Nagendra
Sample obtained from: R. Nagendra

Locality Identifier: KO-DAK ("Kharga Oasis, Dakhla") **Formation:** Dakhla
DT-DAK ("Djebel Ter, Dakhla")

Samples collected here:

- KO-DAK-EXOa: *Exogyra overwegi* (von Buch)
- KO-DAK-EXOb: *Exogyra overwegi* (von Buch)
- DT-DAK-EXOa: *Exogyra overwegi* (von Buch)
- DT-DAK-EXOb: *Exogyra overwegi* (von Buch)

Location: Kharga Oasis region, Egypt
Collection site description: "In green slabs of upper beds of Cretaceous near Christian mummy sepulcher near El Deir, Kharga Oasis, Egypt." Precise locations of El Deir and Djebel Ter outcrops best approximated by the authors at clear outcrops visible in aerial/satellite photography. Sample locality descriptions cross-referenced with Ball (1900) and Tantawy et al. (2001).
Collected by: H.H. Hobbs, Jan. 1913
Sample obtained from: University of Michigan Ruthven Museum of Natural History

Locality Identifier: MK-KAG ("Mount Katmai Quadrangle, Kaguyak") **Formation:** Kaguyak
AF-KAG ("Afognak Quadrangle, Kaguyak")
IL-KAG ("Iliamna Quadrangle, Kaguyak")

Samples collected here:

- MK-KAG-GLYa: *Glycermerita aleuta* (Squires)
- MK-KAG-GLYb: *Glycermerita aleuta* (Squires)
- MK-KAG-GLYc: *Glycermerita aleuta* (Squires)

- MK-KAG-GLYd: *Glycermerita aleuta* (Squires)
- MK-KAG-GLYe: *Glycermerita aleuta* (Squires)
- MK-KAG-BIVa: *Unknown bivalve*
- AF-KAG-GLYa: *Glycermerita aleuta* (Squires)
- IL-KAG-BIVa: *Unknown bivalve*

Location: Mount Katmai region, Alaska Peninsula, Alaska

Collection site description: Coordinates provided by the University of California Museum of Paleontology where available (Berkeley, CA). No additional sampling details available. Associated manuscripts for cross-referencing: Wartes et al. (2013), Peecook and Sidor (2015).

Collected by: Elder, Squires, and Magoon (dates unknown)

Sample obtained from: University of California Museum of Paleontology (Berkeley, CA).

Locality Identifier: OP-PRI ("Ocean Point, Prince Creek") — **Formation:** Prince Creek
EL-SCH ("Elder, Schrader Bluff") — **Formation:** Schrader Bluff

Samples collected here:

- OP-PRI-CYRa: *Cyrtodaria* sp.
- EL-SCH-BIVa: *Unknown bivalve*

Location: North Slope Alaska, OP-PRI: Ocean Point, no additional details available. EL-SCH-BIVa: "On Colville River at center of next bluff E of Umiat Mountain. T1N, R1E, sec. 27, Ctr S line." Locations cross-referenced with Jones and Gryc (1960), Detterman et al. (1963), and Brosgé et al. (1966). Associated manuscripts for cross-referencing and age control: Flores et al. (2007), Flaig and van der Kolk (2015).

Collection site description: Coordinates provided by the University of California Museum of Paleontology where available (Berkeley, CA).

Collected by: L.Marincovich, 1977 (OP-PRI-CYRa); Elder, 1988 (EL-SCH-BIVa)

Sample obtained from: University of California Museum of Paleontology (Berkeley, CA).

Locality Identifier: MC-MOR ("Merced County, Moreno Fm.") — **Formation:** Moreno
MC-GAR ("Merced County, Garzas Sand")

Samples collected here:

- MC-MOR-GLYa: *Glycymeris* sp.
- MC-MOR-GLYb: *Glycymeris* sp.
- MC-MOR-GLYd: *Glycymeris* sp.
- MC-GAR-GRYa: *Gryphaea* sp.
- MC-GAR-GRYb: *Gryphaea* sp.

Location: Merced County, California.

Collection site description: Coordinates provided by the University of California Museum of Paleontology where available (Berkeley, CA). Associated manuscript for cross-referencing and age constraints: Peecook and Sidor (2015).

Collected by: A. Bennison 1937, 1939; L. Briggs 1948

Sample obtained from: University of California Museum of Paleontology (Berkeley, CA).

Locality Identifier: WI-NAN ("Waldron Island") — **Formation:** Northumberland Fm.
SI-NAN ("Sucia Island")

Samples collected here:

- WI-NAN-VANa: *Arca vancouverensis* (Meek)
- SI-NAN-VANa: *Arca vancouverensis* (Meek)

Location: San Juan Islands, Washington

Collection site description: Coordinates provided by the University of California Museum of Paleontology where available (Berkeley, CA), and were sampled from the Late Campanian/Early Maastrichtian Nanaimo

Group, Northumberland Fm. WI-NAN-VANa was retrieved from Wauldron Island and SI-NAN-VANa was retrieved from Sucia Island. Associated manuscripts for cross-referencing: Wartes et al. (2013), Peecook and Sidor (2015).
Collected by: Packard, date unknown (WI-NAN); J.W. Durham, 1948 (SI-NAN)
Sample obtained from: University of California Museum of Paleontology (Berkeley, CA).

Locality Identifier: SC-BAL ("Scania, Balsvik Quarry") **Formation:** Unidentified
Samples collected here:

- SC-BAL-BIVa: *Unknown bivalve*
- SC-BAL-BELa: *Belemnitella* sp.
- SC-BAL-BELb: *Belemnitella* sp.

Location: Scania, Sweden
Collection site description: "Quarry at north end of Ivo Island. Limestone with megafossils." Coordinates provided by the University of California Museum of Paleontology (Berkeley, CA). Associated manuscript for cross-referencing and age control: Christensen (1998).
Collected by: J.T. Gregory 1960
Sample obtained from: University of California Museum of Paleontology (Berkeley, CA).

Locality Identifier: LI-FEZ ("Libya, Fezzan Region") **Formation:** Unknown
Samples collected here:

- LI-FEZ-UNGa: *Agerostrea ungulata* (von Schlotheim)
- SR-CUS-MESa: *Agerostrea ungulata* (von Schlotheim)

Location: Fezzan region, Libya
Collection site description: "According to donor these oysters came from the Fezzan area in Libya. No other data, but kept because of their quality." Coordinates approximated from sample description, and location of outcropping Cretaceous limestones near the road in the Fezzan region by the only significant town/outpost (Murzuq). Sample locality descriptions cross-referenced with Goudarzi (1970) and Ali Falefa El-ghali (2005).
Collected by: Unknown ca. 1967(?)
Sample obtained from: University of California Museum of Paleontology (Berkeley, CA).

Locality Identifier: MOD-PRO ("Modern, Providence") **Formation:** N/A
Samples collected here:

- MOD-PRO-VIRa: *Crassostrea virginica* (Gmelin)
- MOD-PRO-FORa: *Crepidula fornicata* (Linneaus)
- MOD-PRO-DEMa: *Geukensia demissa* (Dillwyn)
- MOD-PRO-BIVa: *Unidentified bivalve*

Location: Sabin Point Park, Providence, Rhode Island
Collection site description: Along the northern shore of the park among the large death assemblage comprising the shoreline.
Collected by: K. Meyer, July 2017

Locality Identifier: MOD-SPE ("Modern, Spectacle Island") **Formation:** N/A
Samples collected here:

- MOD-SPE-VIRa: *Crassostrea virginica* (Gmelin)
- MOD-SPE-VIRb: *Crassostrea virginica* (Gmelin)
- MOD-SPE-VIRc: *Crassostrea virginica* (Gmelin)
- MOD-SPE-VIRd: *Crassostrea virginica* (Gmelin)

Location: Spectacle Island, Boston, Massachusetts
Collection site description: N/A
Collected by: E. Crowther, July 2017

Locality Identifier: MOD-TAH ("Modern, Lake Tahoe") **Formation:** N/A
Samples collected here:
- MOD-TAH-CORa: *Corbicula fluminea* (Müller)
- MOD-TAH-CORb: *Corbicula fluminea* (Müller)
- MOD-TAH-CORc: *Corbicula fluminea* (Müller)
- MOD-TAH-CORd: *Corbicula fluminea* (Müller)

Location: Lake Tahoe, California
Collection site description: N/A
Collected by: I. Winkelstern, 2017

Locality Identifier: MOD-TAH ("Modern, Lake Tahoe") **Formation:** N/A
Samples collected here:
- MOD-TAH-CORa: *Corbicula fluminea* (Müller)
- MOD-TAH-CORb: *Corbicula fluminea* (Müller)
- MOD-TAH-CORc: *Corbicula fluminea* (Müller)
- MOD-TAH-CORd: *Corbicula fluminea* (Müller)

Location: Lake Tahoe, California
Collection site description: N/A
Collected by: I. Winkelstern, 2017

Locality Identifier: MOD-SOU ("Modern, South River") **Formation:** N/A
Samples collected here:
- MOD-SOU-CORa-1.1: *Corbicula fluminea* (Müller)
- MOD-SOU-CORb-1.1: *Corbicula fluminea* (Müller)
- MOD-SOU-CORa-6: *Corbicula fluminea* (Müller)
- MOD-SOU-CORb-6: *Corbicula fluminea* (Müller)
- MOD-SOU-CORa-14: *Corbicula fluminea* (Müller)
- MOD-SOU-CORb-14: *Corbicula fluminea* (Müller)

Location: South River, Virginia
Collection site description: Numbers correspond to relative distance along the South River reach defined by Washburn et al. (2017), where 6 and 14 correspond to approximate downstream locations (precisely 7.8 km and 15.9 km, respectively) and 1.1 is the approximate upstream location (precisely 1.1 km) of the point source of anthropogenic Hg contamination.
Collected by: S. Washburn, 2016

Locality Identifier: PLE-RB ("Pleistocene, Rocky Bay") **Formation:** Rocky Bay
Samples collected here:
- PLE-RB-UNKa: *Unknown bivalve*
- PLE-RB-UNKb: *Unknown bivalve*
- PLE-RB-UNKc: *Unknown bivalve*
- PLE-RB-JAPa: *Cittarium pica* (Linnaeus); Identified as sample RB6 in Winkelstern et al. (2017)

Location: Rocky Bay, Bermuda
Collection site description: Sampling locations detailed in Winkelstern et al. (2017)
Collected by: I. Winkelstern

Locality Identifier: BF-PD ("Burches Ferry") **Formation:** Peedee

Samples collected here:

- BF-PD-BELc: *Belemnitella americana* (d'Orbigny)
- BF-PD-EXOa: *Exogyra costata* (Say)
- BF-PD-EXOe: *Exogyra costata* (Say)

Location: Type section of the Pee Dee Belemnite carbon isotope standard reference material (PDB) at Burches Ferry, South Carolina (Ruffin, 1843; Siple, 1957; Swift, 1966).

Collection site description: Samples were collected from ~6.5 m of exposure of the Peedee formation from three distinct calcareous mudstone horizons bearing a mixed shell hash. Upper contact of the unit is unconformable. Refer to stratigraphic column in Meyer et al. (2018).

Collected by: K.W. Meyer, May 7th, 2015

Sample obtained from: collected by authors

Locality Identifier: ML-CLA ("Moscow Landing (Clayton)") **Formation:** Clayton

Locality Identifier: ML-PRB ("Moscow Landing (Prairie Bluff)") **Formation:** Prairie Bluff

Samples collected here:

Clayton Formation

- ML-CLA-OSTa: *Ostrea pulaskensis* (Harris)
- ML-CLA-OSTb: *Ostrea pulaskensis* (Harris)
- ML-CLA-OSTc: *Ostrea pulaskensis* (Harris)
- ML-CLA-OSTd: *Ostrea pulaskensis* (Harris)

Prairie Bluff Formation

- ML-PRB-EXOa: *Exogyra costata* (Say)
- ML-PRB-EXOb: *Exogyra costata* (Say)
- ML-PRB-EXOc: *Exogyra costata* (Say)
- ML-PRB-EXOd: *Exogyra costata* (Say)
- ML-PRB-EXOe: *Exogyra costata* (Say)
- ML-PRB-EXOf: *Exogyra costata* (Say)
- ML-PRB-EXOg: *Exogyra costata* (Say)
- ML-PRB-EXOh: *Exogyra costata* (Say)
- ML-PRB-EXOi: *Exogyra costata* (Say)

Location: Exposure of the Clayton and Prairie Bluff Formations at Moscow Landing, Alabama.

Collection site description: Clayton samples were collected from three prominent limestone beds (Pine Barren Member) immediately overlying the Prairie Bluff Formation. Prairie Bluff samples were collected across the entire exposure, from the bottom of the section to the base of the large sand channels near the unconformable boundary at the top of the unit. Refer to stratigraphic column found in Meyer et al. (2018).

Collected by: K.W. Meyer, May 14th, 2015

Sample obtained from: collected by authors

Locality Identifier: TR-RIP ("Tombigbee River") **Formation:** Ripley

Samples collected here:

- TR-RIP-EXOa: *Exogyra costata* (Say)
- TR-RIP-EXOb: *Exogyra costata* (Say)
- TR-RIP-EXOc: *Exogyra costata* (Say)
- TR-RIP-EXOd: *Exogyra costata* (Say)

Location: Exposure of the Ripley Formation near Moscow Landing, Alabama, for ~1.5 km along the northern shoreline directly east of where Alabama State Highway 8 crosses the Tombigbee River.

Collection site description: Samples were collected from 4 m of exposure of the Ripley formation below a prominent ~10 cm thick conglomerate layer as an unconformable upper contact on the unit. Refer to stratigraphic column found in Meyer et al. (2018)

Collected by: K.W. Meyer, May 14th, 2015

Sample obtained from: collected by authors

Locality Identifier: MC-PRB ("Marengo County") **Formation:** Prairie Bluff

Samples collected here:

- MC-PRB-EXOa: *Exogyra costata* (Say)
- MC-PRB-EXOb: *Exogyra costata* (Say)

Location: Roadcuts, both sides of Alabama Highway 28, 1.6 miles west of Jefferson, Marengo Co., Alabama (coordinates approximated from sample description, site reexamined by authors on May 14th, 2015 as small roadcut – no new samples were collected) as per Meyer et al. (2018).

Collection site description: GSA 80th Annual Meeting (Alabama Geological Society), p. 66-67

Collected by: D.B. Macurda Jr., Nov. 1967

Sample obtained from: University of Michigan Museum of Paleontology

Locality Identifier: N/A ("Seymour Island") **Formation:** Lopez de Bertodano

Samples collected here:

- E725A-693: *Eselaevitrigonia regina* (Wilckens)
- C772A-708: *Cucullaea antarctica* (Wilckens)
- C1109A-855: *Cucullaea antarctica* (Wilckens)
- L1609-887: *Lahillia larseni* (Sharman & Newton)
- C1556-838: *Cucullaea antarctica* (Wilckens)
- L776A-925: *Lahillia larseni* (Sharman & Newton)
- E757-1-925: *Eselaevitrigonia regina* (Wilckens)
- L1480-973: *Lahillia larseni* (Sharman & Newton)
- L1474-1016: *Lahillia larseni* (Sharman & Newton)
- C1517A-1025: *Cucullaea antarctica* (Wilckens)
- C1184-1032: *Cucullaea antarctica* (Wilckens)
- L1161-1051: *Lahillia larseni* (Sharman & Newton)
- L477D-1058: *Lahillia larseni* (Sharman & Newton)
- L1529C-1059: *Lahillia larseni* (Sharman & Newton)
- L1430-1098: *Lahillia larseni* (Sharman & Newton)

Location: Exposed areas of Seymour Island, Antarctica

Collection site description: various locations, site ID indicated in sample name prior to hyphen, excluding first letter which indicates species. Plane projected to various stratigraphic heights, indicated in sample names following the hyphen.

Collected by: W.J. Zinsmeister over multiple field seasons in the 80's and 90's.

Sample obtained from: Zinsmeister, as part of a PhD thesis project of Andrea Dutton in the early 2000's. Kept at UM since in the possession of K.C. Lohmann.

Supplementary Note 2. *Sample preservation regarding [Hg], Δ_{47}, and $^{87}Sr/^{86}Sr$ compositions*

The material we used in this study displays exceptional preservation for both carbonate clumped isotopes (Petersen et al., 2016; Meyer et al., 2018) as well as for the determination of [Hg]. The mercury preserved in these specimens appears to be of primary origin, and is likely the product of direct, but disordered substitution as inorganic Hg(II). Ahmad et al. (2013) support this interpretation through the observation of defects in shell organic matrix seen in scanning and transmission electron microscopy of modern bivalves from Hg contaminated sites in Portugal, but the mechanism for how Hg is incorporated into mollusk shells is unknown (either by direct incorporation in the shell carbonate or entrapment in the shell organic matrix).

In this study, we have relied on Δ_{47} and $^{87}Sr/^{86}Sr$ values for determining coastal marine temperatures and approximate age relationships, respectively. We also used both of these isotopic compositions as our primary means of evaluating the potential for diagenetic alteration of carbonate in analyzed specimens, which could also be indicators for diagenetic influence on [Hg] values. Measured $^{87}Sr/^{86}Sr$ values for these samples were compared to values predicted with the LOWESS model (McArthur et al., 2001; McArthur, pers. comm., 2015). Samples with $^{87}Sr/^{86}Sr$ values outside the range given by the LOWESS curve for the Late Campanian through the Late Maastrichtian were thought to be either influenced by enhanced terrigenous inputs (e.g. in estuarine environments), or by diagenetic alteration (as described by Bryant et al., 1995). Eight samples exhibited anomalous $^{87}Sr/^{86}Sr$ values, which implied these shells were recording a non-marine and/or diagenetic environment. In all of these cases, where Δ_{47} was also measured, the Δ_{47} values supported an interpretation of thermal alteration, as defined by elevated Δ_{47}-derived temperatures of a sample region (temperatures ≥ 35 °C globally, or temperatures ≥ 20 °C collected from latitudes ≥ 50 °N; please refer to Supplementary Table S4). In the seven instances

where both Δ_{47} and $^{87}Sr/^{86}Sr$ values suggested possible alteration, samples were classified as 'altered' and excluded from interpretations of [Hg]; this comprised all specimens from the Mt. Katmai and San Juan Islands regions. These regions represent parts of the accreted Wrangellia and Peninsular Terranes of western North America (Jones et al., 1977; Greene et al., 2005) and have been subjected to intense deformational tectonic histories (Flores et al., 2007; Wartes et al., 2012; Peecook and Sidor, 2015; Flaig and van der Kolk, 2015), compared to all other sample regions considered in this study with comparatively passive tectonic settings and relatively shallow burial histories (Ball, 1900; Goudarzi, 1970; Christensen, 1998; Tantawy et al., 2001; Ali Kalefa El-ghali, 2005; Nagendra et al., 2011; Zakharov et al., 2011; Petersen et al., 2016; de Winter et al., 2017). It is possible that a sample may be altered in terms of clumped isotope composition (by solid-state bond reordering), but not in terms of $^{87}Sr/^{86}Sr$ and/or [Hg] values. In many respects, the most sensitive indicators of thermal overprinting or diagenetic alteration in a biogenic carbonate is the Δ_{47} composition of a given sample followed by potential low-temperature overprinting of $^{87}Sr/^{86}Sr$ (e.g. Bryant et al., 1995), whereas influences of diagenesis on [Hg] are presently unknown.

In making a first attempt at evaluating the role of diagenetic influences on [Hg] in biogenic carbonates, we determined [Hg] in Carrara marble. Carrara marble is a common geologic reference material that has been extensively used as a traditional $\delta^{18}O$ and $\delta^{13}C$ isotope standard. The boiling point of metallic Hg is 357 °C (Lide, 2004), and is readily capable of volatilizing at surface temperatures (e.g. evaporation from soils; Schlüter, 2000). Alternatively, Hg(II)-bearing phases such as mercury sulfides (HgS) and mercury oxides (HgO) have been demonstrated to have peak thermal decomposition temperatures (at surface pressures) of between 360 to 470 °C and 470 to 500 °C, respectively (Leckey and Nulf, 1994; Baláž and Godočikoyá,

2001). It has been suggested that Carrara marble has experienced peak metamorphic temperatures of 430 to 450 °C (Leiss and Molli, 2003) and fluid alteration at approximately 400 °C (Costagliola et al., 1999). Given the constraints on the metamorphic history of Carrara marble, we would not expect any metallic Hg^0 to be present in these samples, and only small quantities of Hg(II) mineral phases. We determined [Hg] in Carrara marble to be 1.4 ng g^{-1}, significantly elevated above average analytical blank values of 0.0079 ± 0.021 ng g^{-1}, but only just outside the 'Pre-Industrial background' level. The retention of Hg in Carrara marble is encouraging for evaluating Hg in carbonates of various origins throughout the geological record given the possibility for Hg retention despite experiencing elevated metamorphic temperatures/conditions. For the samples of biogenic carbonate measured for [Hg] in this study, there is only minor concern of [Hg] values being altered (based on Δ_{47} and $^{87}Sr/^{86}Sr$ compositions with the former limiting burial temperatures to ≤ 100 °C), but we acknowledge the need for Hg-specific screening methods for diagenetic alteration.

At present, we know of only one study that has quantified (via inductively-coupled plasma mass spectrometry) and documented fossilized biomineral replacement with cinnabar (HgS) filling-in the porous structure of rodent dentition, however this is acknowledged by the authors as exceedingly rare and the consequence of a nearby cinnabar deposits to the fossil locality (García-Alix et al., 2012). Additionally, only two recorded accounts of abiotic mercury carbonates have ever been documented, the polymorphs peterbaylissite and clearcreekite, $Hg_3^{1+}(CO_3)(OH)\cdot 2H_2O$ (Roberts et al., 1995, 2001). Both minerals were discovered in the Clear Creek mercury mine of the New Idria district in San Benito County (California) and are described as being extremely rare and found in either immediate proximity to or co-occurring with cinnabar or native mercury (Roberts et al., 1995, 2001). Therefore, we believe that, while

possible, Hg diagenetic influence on marine carbonates would more likely result in the loss of Hg from a specimen with the exception of instances where native mercury or cinnabar deposits may be in close proximity to fossil deposits (none of our sites, to our knowledge).

Supplementary References

1. Ahmad, I. *et al.* Morphological, compositional and ultrastructural changes in the Scrobicularia plana shell in response to environmental mercury – An indelible fingerprint of metal exposure? *Chemosphere* **90,** 2697–2704 (2013).

2. Ali Kalefa El-ghali, M. Depositional environments and sequence stratigraphy of paralic glacial, paraglacial and postglacial Upper Ordovician siliciclastic deposits in the Murzuq Basin, SW Libya. *Sedimentary Geology* **177,** 145–173 (2005).

3. Baláž, P. & Godočiková, E. Thermal Reduction of Mechanically Activated Cinnabar (HgS) and Stibnite (Sb2S3). *Journal of Thermal Analysis and Calorimetry* **65,** 51–57 (2001).

4. Ball, J. *Kharga Oasis: Its Topography and Geology*. (Cairo National Printing Department, 1900).

5. Brosgé, W. P., Whittington, C. L. & Morris, R. H. *Geology of the Umiat-Maybe Creek Region, Alaska. USGS Professional Paper* **303-H,** (1966).

6. Bryant, J. D., Jones, D. S. & Mueller, P. A. Influence of freshwater flux on 87Sr/86Sr chronostratigraphy in marginal marine environments and dating of vertebrate and invertebrate faunas. *Journal of Paleontology* **69,** 1–6 (1995).

7. Christensen, W. K. Belemnitella from the lowermost Maastrichtian of Scania, southern Sweden. *Bulletin of the Geological Society of Denmark* **45,** 1–12 (1998).

8. Costagliola, P., Benvenuti, M., Maineri, C., Lattanzi, P. & Ruggieri, G. Fluid circulation in the Apuane Alps core complex: evidence from extension veins in the Carrara marble. *Mineralogical Magazine* **63,** 111–111 (1999).

9. Detterman, R. L., Bickel, R. S. & Gryc, G. *Geology of the Chandler River Region, Alaska. USGS Professional Paper* **303-E,** 102 (1963).

10. de Winter, N. J. *et al.* An assessment of latest Cretaceous *Pycnodonte vesicularis* (Lamarck, 1806) shells as records for palaeoseasonality: A multi-proxy investigation. *Clim. Past Discuss.* 1–36 (2017). doi:10.5194/cp-2017-120

11. Dickin, A. P. *Radiogenic Isotope Geology 2nd (second) edition published by Cambridge University Press [Paperback] (2005)*. (Cambridge University Press, 2005).

12. Flaig, P. P. & van der Kolk, D. A. Depositional Environments of the Prince Creek Formation Along the East Side of the Toolik Rover, Sagavanirktok Quadrangle, North Slope, Alaska. *State of Alaska Department of Natural Resources Division of Geological &Geophysical Surveys Preliminary Interpretive Report 2015-4* (2015). doi:10.14509/29407

13. Flores, R. M. *et al.* Stratigraphy and Facies of Cretaceous Schrader Bluff and Prince Creek Formations in Colville River Bluffs, North Slope, Alaska. *USGS Professional Paper* **1748,** 45 (2007).

14. García-Alix, A., Minwer-Barakat, R., Martín Suárez, E., Freudenthal, M. & Delgado Huertas, A. (2012) Cinnabar mineralization in fossil small mammal remains as a consequence of diagenetic processes. *Lethaia* **46,** 1–6.

15. Goudarzi, G. H. Geology and mineral resources of Libya : a reconnaissance. *USGS Professional Paper* **660,** (1970).

16. Greene, A. R., Scoates, J. S. & Weis, D. Wrangellia Terrane on Vancouver Island, British Columbia: Distribution of Flood Basalts with Implications for Potential Ni-Cu-PGE Mineralization in Southwestern British Columbia. *British Columbia Geological Survey, Geological Fieldwork Paper* - 1–12 (2005).

17. Jones, D. L. & Gryc, G. *Upper Cretaceous Pelecypods of the Genus Inoceramus from Northern Alaska. USGS Professional Paper* **334-E,** 17 (1960).

18. Jones, D. L., Silberling, N. J. & Hillhouse, J. Wrangellia—A displaced terrane in northwestern North America. *Canadian Journal of Earth Sciences* **14,** 2565–2577 (1977).

19. Leckey, J. H. & Nulf, L. E. *Thermal decomposition of mercuric sulfide*. *Oak Ridge Technical Report Y/DZ-1124* (1994). doi:10.2172/41313

20. Leiss, B. & Molli, G. 'High-temperature' texture in naturally deformed Carrara marble from the Alpi Apuane, Italy. *Journal of Structural Geology* **25,** 649–658 (2003).

21. Lide, D. R. *CRC Handbook of Chemistry and Physics, 85th Edition*. (CRC Press, 2004).

22. McArthur, J. M., Howarth, R. J. & Bailey, T. R. Strontium isotope stratigraphy: LOWESS version 3: Best fit to the marine Sr-isotope curve for 0-509 Ma and accompanying look-up table for deriving numerical age. *Journal of Geology* **109,** 155–170 (2001).

23. Meyer, K. W., Petersen, S. V., Lohmann, K. C. & Winkelstern, I. Z. Climate of the Late Cretaceous North American Gulf and Atlantic Coasts. *Cretaceous Research* **89,** 160–173 (2018).

24. Nagendra, R. *et al.* Sequence surfaces and paleobathymetric trends in Albian to Maastrichtian sediments of Ariyalur area, Cauvery Basin, India. *Marine and Petroleum Geology* **28,** 895–905 (2011).

25. Peecook, B. R. & Sidor, C. A. The First Dinosaur from Washington State and a Review of Pacific Coast Dinosaurs from North America. *PLoS ONE* **10,** e0127792–15 (2015).

26. Petersen, S. V. *et al.* Temperature and salinity of the Late Cretaceous Western Interior Seaway. *Geology* G38311.1–4 (2016). doi:10.1130/G38311.1

27. Roberts, A. C. *et al.* Peterbaylissite, Hg (super 1+) 3 (CO 3)(OH).2H 2 O, a new mineral species from the Clear Creek Claim, San Benito County, California. *The Canadian Mineralogist* **33,** 47–53 (1995).

28. Roberts, A. C. *et al.* Clearcreekite, A New Polymorph of Hg1+3(CO3)(OH)2H2O, from the Clear Creek Claim, San Benito County, California. *The Canadian Mineralogist* **39,** 779–784 (2001).

29. Ruffin, E., (1843). Report on the Commencement and Progress of the Agricultural Survey of South Carolina, p. 1–194.

30. Schlüter, K. Review: evaporation of mercury from soils. An integration and synthesis of current knowledge. *Environmental Geology* **39,** 249–271 (2000).

31. Tantawy, A. A. *et al.* Maastrichtian to Paleocene depositional environment of the Dakhla Formation, Western Desert, Egypt: sedimentology, mineralogy, and integrated micro- and macrofossil biostratigraphies. *Cretaceous Research* **22,** 795–827 (2001).

32. Wartes, M. A. *et al.* Preliminary stratigraphy and facies analysis of the Upper Cretaceous Kaguyak Formation, including a brief summary of newly discovered oil stain, upper Alaska Peninsula. *Overview of 2012 field studies: Upper Alaska Peninsula and west side of lower Cook Inlet, Alaska* 25–32 (2012). doi:10.14509/24849

33. Washburn S. J., Blum J. D., Demers J. D., Kurz A. Y. and Landis R. C. (2017) Isotopic Characterization of Mercury Downstream of Historic Industrial Contamination in the South River, Virginia. *Environ. Sci. Technol.* **51**, 10965–10973.

34. Washburn, S. J., Blum, J. D., Kurz, A. Y. & Pizzuto, J. E. Spatial and temporal variation in the isotopic composition of mercury in the South River, VA. *Chemical Geology* **494,** 96–108 (2018).

35. Woelders, L. *et al.* Latest Cretaceous climatic and environmental change in the South Atlantic region. *Paleoceanography* **32,** 466–483 (2017).

36. Winkelstern I. Z., Rowe M. P., Lohmann K. C., Defliese W. F., Petersen S. V. and Brewer A. W. (2017) Meltwater pulse recorded in Last Interglacial mollusk shells from Bermuda. *Paleoceanography* **32**, 132–145.

37. Zakharov, Y. D. *et al.* Cretaceous climate oscillations in the southern palaeolatitudes: New stable isotope evidence from India and Madagascar. *Cretaceous Research* **32,** 623–645 (2011).
