# Supplementary material for: Biogenic carbonate mercury and marine temperature records reveal global influence of Late Cretaceous Deccan Traps
Source: Nat Commun. 2019 Dec 16;10:5356. doi: 10.1038/s41467-019-13366-0 (PMC6915775; doi:10.1038/s41467-019-13366-0)
Supplement: Supplementary file 3 — Description of Additional Supplementary Files [file 41467_2019_13366_MOESM3_ESM.pdf]

Description of Additional Supplementary Files

File Name: Supplementary Data 1

Description: Strontium isotopic results (also found in Supplementary Table 1)

File Name: Supplementary Data 2

Description: Mercury concentration results (also found in Supplementary Table 2)

File Name: Supplementary Data 3

Description: Measured oxygen, carbon, and clumped isotopic results – δ18O, δ13C, and Δ47 (also found in Supplementary Table 3)
